# Repeat surgical interventions in non-Fuchs corneal dystrophies: insights from eight decades of histopathological and clinical data

**DOI:** 10.1038/s41433-026-04867-6

**Published:** 2026-09-22

**Authors:** Othmane Touirssa, Philip Maier, Daniel Boehringer, Claudia Auw-Haedrich, Mateusz Glegola, Thomas Reinhard, Simone Nuessle

**Affiliations:** 1 Eye Centre, University Medical Centre, Albert-Ludwigs-University of Freiburg, Freiburg, Germany; 2 Eye Clinic, University Hospital Basel, Basel, Switzerland

**Keywords:** Corneal diseases, Hereditary eye disease, Eye manifestations, Vision disorders, Eye abnormalities

## Abstract

**Background/Objectives:**

To evaluate the proportion of specimens and patients linked to surgical re-interventions and the types of preceding procedures in non-Fuchs corneal dystrophies using an eight-decade dataset.

**Subjects/Methods:**

A total of 372 histopathological specimens with confirmed diagnoses of non-Fuchs corneal dystrophies, collected between 1945 and 2024, were retrospectively examined with associated clinical data. The proportion of re-interventions was assessed on both a per-specimen and per-patient basis by dystrophy type. For each of the 94 re-intervention specimens, the type of prior procedure leading to re-intervention, including manual superficial keratectomy (MSK), phototherapeutic keratectomy (PTK), penetrating keratoplasty (PKP) and limbo-keratoplasty (LimboKP), was analysed.

**Results:**

Gelatinous drop-like dystrophy (GDLD) showed the highest proportion of specimens obtained after surgical re-intervention (86.2%), including specimens following LimboKP (13/25). Granular dystrophy (GCD) and lattice dystrophy (LCD) also accounted for high proportions of re-intervention specimens (39.2% and 32.8%, respectively), most following PKP (16/31 and 17/21, respectively). In contrast, macular corneal dystrophy (MCD), showed a low proportion of re-interventions per specimen (4.6%) with all specimens linked to minimally invasive techniques. No re-interventions were observed in endothelial dystrophies.

**Conclusions:**

Surgical re-intervention is frequent in epithelial and epithelial-stromal dystrophies, particularly GDLD, GCD and LCD, and may occur even after targeted procedures like LimboKP. In contrast, stromal dystrophies like MCD rarely required repeat surgery, especially following PKP. These findings highlight the limitations of current surgical approaches in recurrence-prone dystrophies and support the need for dystrophy-specific strategies, including the development of improved techniques to reduce recurrence and the implementation of risk-adapted follow-up.

## Introduction

Corneal dystrophies represent a heterogeneous group of inherited disorders affecting corneal transparency and visual function [1]. While Fuchs endothelial corneal dystrophy (FECD) is the most frequent form [2], non-Fuchs corneal dystrophies encompass a wide range of less common conditions with diverse anatomical localisation and clinical presentations [3]. The IC3D classification system (International Committee for the Classification of Corneal Dystrophies) provides a standardised framework for categorising these dystrophies based on anatomical localisation, as well as clinical, histopathological, and genetic characteristics [4].

Surgical intervention is frequently required in progressive, symptomatic or advanced cases, with the choice of technique depending on the extent and location of the corneal changes. Options range from superficial ablations, such as phototherapeutic keratectomy (PTK), commonly employed in anterior dystrophies, to full-thickness procedures like penetrating keratoplasty (PKP) for deeper stromal disease [5]. A more invasive procedure that requires systemic immunosuppression is limbo-keratoplasty (LimboKP), a surgical technique involving the transplantation of a donor graft containing a portion of the limbus, introduced in 1993 by Sundmacher et al. [6]. Despite these surgical options, recurrence or progression remain a major challenge in certain dystrophy types. Epithelial and epithelial-stromal dystrophies are particularly prone to recurrence [4], whereas stromal and rarely endothelial dystrophies tend to recur less frequently [7, 8]. These risks are influenced not only by the dystrophy type but also by the chosen surgical method in regard of the anatomical localisation of the diseased tissue [9, 10]. While recurrence following surgery has been documented in various types, systematic data on the need for surgical re-intervention remain limited, especially across long-term observational cohorts. Most studies on recurrence are clinically driven and focus on a single dystrophy type, evaluating recurrence rates based on postoperative findings. Differences in recurrence risk between dystrophy types are often assumed but not extensively quantified across surgical methods.

In contrast, the present study applies a unique, reverse-perspective approach by analysing a comprehensive histopathological archive spanning eight decades while focusing exclusively on specimens obtained through surgical re-intervention. This approach not only enables us to characterise the distribution of dystrophies associated with repeat surgery, which complements clinical recurrence studies, but also helps identify surgical techniques potentially associated with a higher likelihood of subsequent intervention. We therefore aim to provide an additional descriptive perspective on re-interventions across dystrophy types and preceding surgical procedures in these rare conditions.

## Materials and methods

### Study design

This retrospective observational study builds on a previously published analysis from our group that characterised the distribution and surgical management of corneal dystrophies over an eight-decade period between January 1945 and December 2024 at the Eye Centre, Medical Centre, University of Freiburg [11]. That study primarily focused on the overall distribution of dystrophy types, their temporal trends, and the specific surgical procedures used to obtain the histopathological specimens. The diagnostic framework and ethical standards for the present investigation are consistent with the antecedent study. The 3rd Edition of the IC3D classification was used for both analyses. Although keratoconus is considered a dystrophy by some [12], it is not included in the IC3D classification, and therefore, cases of keratoconus are not included in the dataset. This study adhered to the principles of the Declaration of Helsinki and received approval from the local ethics committee (Ethics Committee at the University of Freiburg Medical Centre, approval number 25-1067-S1-retro), which granted a waiver of informed consent.

### Re-intervention subset

In contrast, the present study addresses a distinct research question by focusing exclusively on surgical re-interventions within the non-Fuchs dataset. The objectives were twofold: first, to determine the proportion of re-interventions for each dystrophy type on both a per-specimen and per-patient basis, and second, to analyse the preceding surgical procedures that led to the re-intervention specimens. For this purpose, a systematic review of the records for all 372 non-Fuchs specimens was performed to identify the re-intervention subset, which comprised 94 specimens. A specimen was included in this subset if its accompanying histopathological or medical records documented at least one prior ipsilateral corneal surgical procedure for the same underlying corneal dystrophy. In all included specimens, the submitted tissue showed specific dystrophic deposits or changes characteristic of the underlying corneal dystrophy.

For each specimen in this subset, the preceding procedure was identified. This was defined as the most recent surgery prior to the intervention that produced the specimen, as determined through a review of all available records. The identified procedures were then categorised as: manual superficial keratectomy (MSK), phototherapeutic keratectomy (PTK), penetrating keratoplasty (PKP), limbo-keratoplasty (LimboKP), or Descemet Membrane Endothelial Keratoplasty (DMEK). No cases in this series were managed with Deep Anterior Lamellar Keratoplasty (DALK) or Descemet Stripping Automated Endothelial Keratoplasty (DSAEK).

### Statistical analysis

All data were analysed using descriptive statistics. No formal sample size calculation was performed because this was a retrospective archive-based study including all non-Fuchs corneal dystrophy specimens identified during the study period. Randomisation and blinding were not applicable. The main outcomes were the proportion of cases linked to re-intervention, which was analysed on two distinct levels, per-specimen and per-patient and the distribution of preceding surgical procedures for the re-intervention specimens, stratified by dystrophy type. The per-specimen proportion was calculated as the number of specimens derived from a re-intervention divided by the total number of specimens for that dystrophy type. The per-patient proportion was calculated as the number of unique patients who underwent at least one re-intervention divided by the total number of unique patients diagnosed with that dystrophy type. Additional descriptive analyses were performed for the re-intervention subset, including patient age at the first archived re-intervention specimen, sex distribution, number of affected eyes, ocular laterality, bilateral involvement, and temporal spacing between successive archived same-eye re-intervention specimens. Continuous variables are reported as mean ± standard deviation, median, and range, where appropriate. Categorical variables are reported as counts and percentages. All data processing, statistical analyses, and visualisations were conducted using Python within Visual Studio Code (Version: 1.102.1) on macOS. Due to the archival nature of the data, follow-up durations and time intervals between surgeries were inconsistently available, particularly in older or externally referred cases, precluding survival analysis.

## Results

Of the 372 specimens from patients with non-Fuchs dystrophies, 94 (25.3%) were obtained following surgical re-intervention, corresponding to 58 unique patients and 69 eyes. All 94 specimens were linked to a documented prior surgical intervention in the same eye for the same underlying dystrophy. The specimens included 43 from right eyes (45.7%) and 51 from left eyes (54.3%). Overall, 47 patients contributed re-intervention specimens from one eye only (81.0%), whereas 11 patients had bilateral re-intervention specimens (19.0%).

Among the 58 patients with histopathologically documented re-intervention specimens, the mean age at the first surgical re-intervention was 54.6 ± 16.1 years, and 28 patients were female (48.3%) and 30 were male (51.7%).

Temporal spacing between archived same-eye re-intervention specimens was assessed for all eyes contributing more than one specimen from the same eye (*n* = 13 eyes). Across 25 consecutive intervals between successive same-eye specimens, the median interval was 4.0 years, with a mean interval of 4.2 ± 3.6 years and a range of 0.8–18.7 years.

**Fig. 1:**
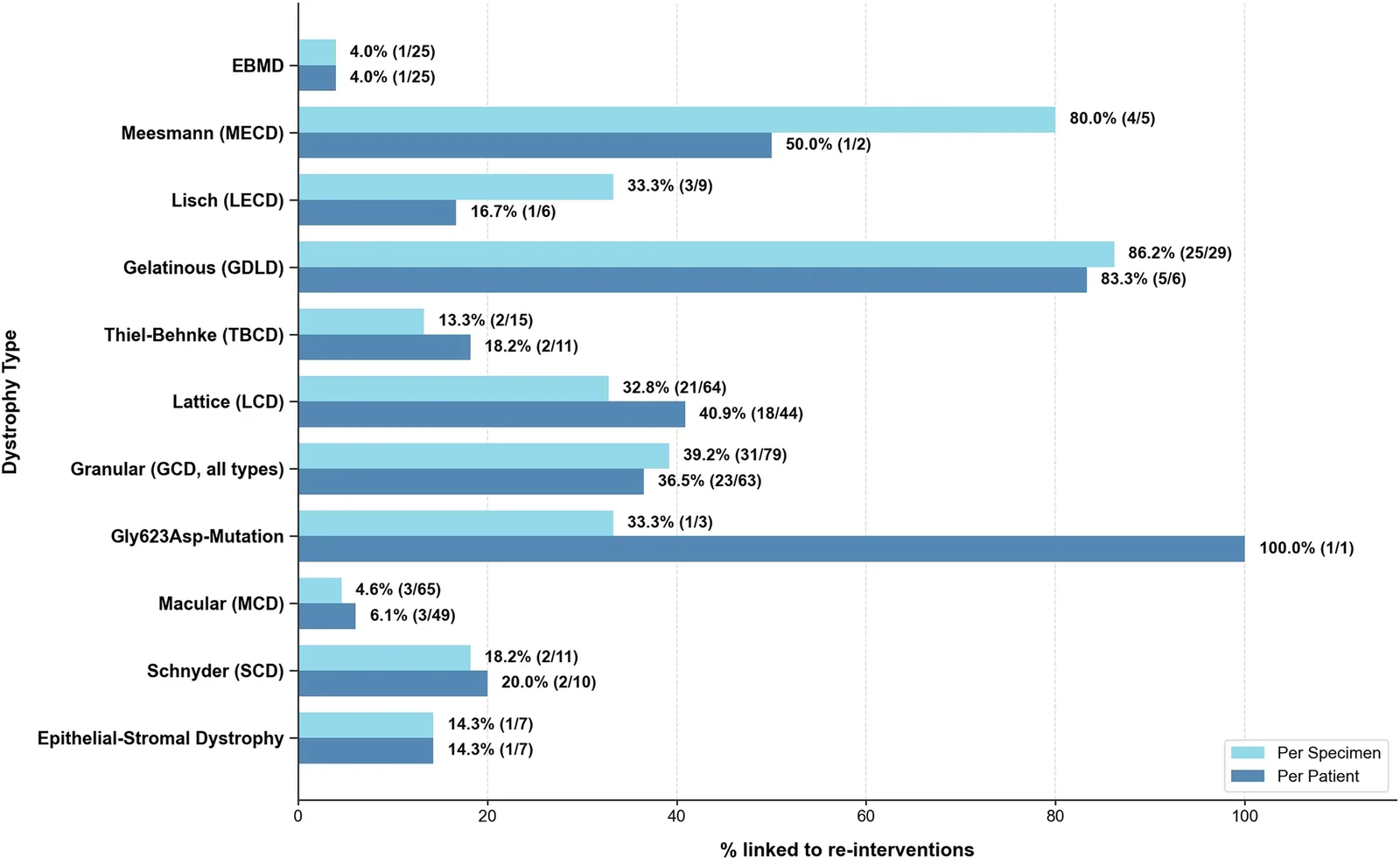
Proportion of surgical re-interventions for non-Fuchs corneal dystrophies, stratified by corneal dystrophy type, analysed per specimen and per patient. Each dystrophy type is represented by two bars: the light blue bar shows the percentage of specimens obtained from a re-intervention out of the total specimens for that dystrophy, while the dark blue bar shows the percentage of patients who underwent re-intervention out of the total patients for that dystrophy. The adjacent labels provide the exact percentage and the underlying counts (re-interventions/total). Dystrophy types without documented re-interventions are not displayed. EBMD Epithelial basement membrane dystrophy, MECD Meesmann corneal dystrophy, LECD Lisch epithelial corneal dystrophy, GDLD Gelatinous drop-like corneal dystrophy, TBCD Thiel–Behnke corneal dystrophy, LCD Lattice corneal dystrophy, GCD Granular corneal dystrophy, Gly glycine, Asp aspartic acid, Gly623Asp represents a corneal dystrophy caused by a G-to-A mutation in exon 14 of the *TGFBI* gene, resulting in substitution of glycine with aspartic acid at codon 623, MCD Macular corneal dystrophy, SCD Schnyder corneal dystrophy, Epithelial-stromal Dystrophy, unspecified epithelial-stromal corneal dystrophy.

**Fig. 2:**
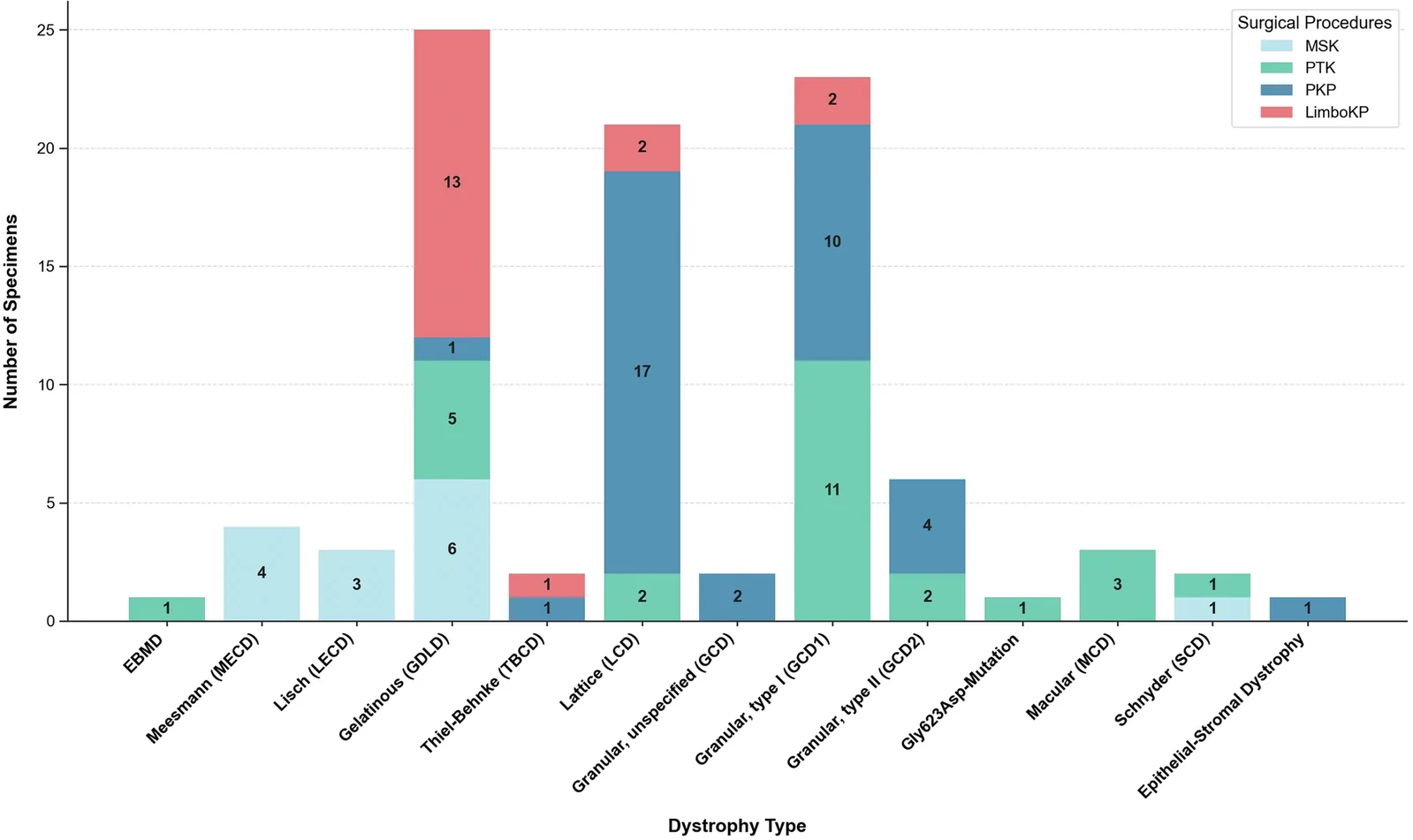
Surgical procedures prior to re-interventions per specimen, grouped by corneal dystrophy type. EBMD Epithelial basement membrane dystrophy, MECD Meesmann corneal dystrophy, LECD Lisch epithelial corneal dystrophy, GDLD Gelatinous drop-like corneal dystrophy, TBCD Thiel–Behnke corneal dystrophy, LCD Lattice corneal dystrophy, GCD Granular corneal dystrophy, Gly glycine, Asp aspartic acid, Gly623Asp represents a corneal dystrophy caused by a G-to-A mutation in exon 14 of the *TGFBI* gene, leading to the substitution of glycine with aspartic acid at amino acid position 623, MCD Macular corneal dystrophy, SCD Schnyder corneal dystrophy, MSK manual superficial keratectomy, PTK phototherapeutic keratectomy, PKP penetrating keratoplasty, LimboKP limbo-keratoplasty.

Table 1 details re-intervention specimens and patients by dystrophy type, including age, sex, affected eyes, ocular laterality, bilateral involvement, and individual consecutive time intervals between same-eye surgical re-interventions. Figures 1 and 2 illustrate the proportion of histopathological specimens derived from cases with documented surgical re-interventions across different dystrophy types. Figure 1 shows the proportion of re-interventions calculated both per specimen and per patient for each dystrophy type, while Fig. 2 presents the preceding surgical procedures performed prior to re-intervention per specimen.

**Table 1:** Patient-level characteristics and temporal spacing of re-intervention specimens by dystrophy type (*n* = 94 specimens, 69 eyes, 58 patients).

Dystrophy type	Patients (bilateral)^a^	Age at 1st re-intervention (y)^b^	Sex (f/m)^c^	Number of eyes	OD specimens	OS specimens
**EBMD**	**1 (0)**	**82.0**	**0 f/1 m**	**1**	**0**	**1**
**Meesmann (MECD)**	**1 (1)**	**40.0**	**1 f/0 m**	**2**	**2**	**2**
	*Patient 1*	*40.0 (OD: 5.1; OS: 4.0)*	*f*	2	2	2
**Lisch (LECD)**	**1 (0)**	**57.0**	**0 f/1 m**	**1**	**3**^**d**^	**0**
**Gelatinous (GDLD)**	**5 (4)**	**32.2** ± **10.3**	**3 f/2 m**	**9**	**14**	**11**
	*Patient 2*	*40.0 (OS: 1.0)*	*m*	2	1	2
	*Patient 3*	*29.0 (OD: 6.1/0.8/2.5/6.0; OS: 4.0)*	*f*	2	5	2
	*Patient 4*	*28.0 (OD: 6.6/3.1/1.0/4.0; OS: 4.6/5.0)*	*f*	2	5	3
	*Patient 5*	*19.0 (OD: 6.6; OS: 6.1/0.8/6.1)*	*f*	2	2	4
**Thiel–Behnke (TBCD)**	**2 (0)**	**52.5** ± **7.8**	**2 f/0 m**	**2**	**1**	**1**
**Lattice (LCD)**	**18 (2)**	**61.0** ± **10.2**	**11 f/7 m**	**20**	**7**	**14**^**d**^
	*Patient 6*	*56.0*	*f*	2	1	1
	*Patient 7*	*42.0*	*m*	2	1	1
**Granular, unspecified (GCD)**	**2 (0)**	**42.0** ± **7.1**	**1 f/1 m**	**2**	**1**	**1**
**Granular, type I (GCD1)**	**16 (3)**	**55.1** ± **19.8**	**7 f/9 m**	**19**	**9**	**14**
	*Patient 8*	*48.0 (OD: 5.0/1.4; OS: 1.4/2.1)*	*m*	2	3	3
	*Patient 9*	*56.0*	*f*	2	1	1
	*Patient 10*	*70.0*	*f*	2	1	1
**Granular, type II (GCD2)**	**5 (1)**	**58.8** ± **5.8**	**1 f/4 m**	**6**	**3**	**3**
	*Patient 11*	*59.0*	*m*	2	1	1
**Gly623Asp-dystrophy**	**1 (0)**	**68.0**	**0 f/1 m**	**1**	**0**	**1**
**Macular (MCD)**	**3 (0)**	**35.3** ± **12.1**	**1 f/2 m**	**3**	**2**	**1**
**Schnyder (SCD)**	**2 (0)**	**61.0** ± **0.0**	**0 f/2 m**	**2**	**1**	**1**
**Epithelial-Stromal Dystrophy**	**1 (0)**	**66.0**	**1 f/0 m**	**1**	**0**	**1**
**Total**	**58 (11)**	**54.6** ± **16.1**	**28 f/30 m**	**69**	**43 (45.7%)**	**51 (54.3%)**

*OD* oculus dexter, right eye, *OS* oculus sinister, left eye, *y* years, *f* female, *m* male, *EBMD* Epithelial basement membrane dystrophy, *MECD* Meesmann corneal dystrophy, *LECD* Lisch epithelial corneal dystrophy, *GDLD* Gelatinous drop-like corneal dystrophy, *TBCD* Thiel–Behnke corneal dystrophy, *LCD* Lattice corneal dystrophy, *GCD* Granular corneal dystrophy, *Gly* glycine, *Asp* aspartic acid, *Gly623Asp-dystrophy* represents a corneal dystrophy caused by a G-to-A mutation in exon 14 of the *TGFBI* gene, leading to the substitution of glycine with aspartic acid at amino acid position 623, *MCD* Macular corneal dystrophy, *SCD* Schnyder corneal dystrophy, *Epithelial-stromal Dystrophy* unspecified epithelial-stromal corneal dystrophy. Other non-Fuchs corneal dystrophies were analysed but are omitted from Table 1 due to no surgical re-interventions. Bold text and values denote dystrophy-level summary rows and the overall total row; non-bold patient sub-rows represent individual patients with bilateral re-intervention specimens.

^a^Presented as: Number of patients with re-intervention specimens (number of those patients with bilateral re-intervention specimens). Unilateral patients can be calculated as the difference. Patients 1–11 represent the 11 patients with bilateral re-intervention specimens, shown as nested sub-rows under each specific dystrophy type.

^b^For main rows with more than one patient, values represent the mean age ± standard deviation at the first archived re-intervention specimen. For rows with one patient, the individual age is shown. For patient sub-rows (Patients 1–11), values represent the individual’s age at first re-intervention specimen, with consecutive time intervals (in years) between successive archived same-eye re-intervention specimens shown in parentheses chronologically separated by slashes (e.g., OD: 5.0/1.4 indicates consecutive intervals of 5.0 years between the first and second, and 1.4 years between the second and third archived specimens on the right eye).

^c^Sex assigned at birth presented as: Number of females/Number of males (calculated strictly on a per-patient basis).

^d^Additionally, two unilateral patients with re-intervention specimens from a single eye had multiple archived same-eye specimens: one patient with LECD (OD, age 57, male, intervals of 1.2/2.0 y) and one patient with LCD (OS, age 52, male, interval of 18.7 y).

### Proportion of surgical re-interventions

The proportion of specimens obtained following surgical re-intervention varied strongly among dystrophy types, with the analysis performed on both a per-specimen and per-patient basis, as detailed in Fig. 1. Gelatinous drop-like dystrophy (GDLD) and Meesmann corneal dystrophy (MECD) exhibited the highest proportions based on specimens. For GDLD, 25/29 specimens (86.2%) were derived from re-interventions, this corresponded to 5 of 6 patients (83.3%) requiring repeat surgery. For MECD, the specimen-based proportion was 4 of 5 (80.0%); however, as these re-interventions occurred in a single individual, the patient-based proportion was one of 2 patients (50.0%). A substantial frequency of re-intervention was also noted for granular corneal dystrophy (GCD, all types combined), with 31/79 specimens (39.2%) and 23 of 63 patients (36.5%) being linked to re-interventions. Within the GCD group, frequencies varied by subtype: Granular corneal dystrophy, type 1 (GCD1) accounted for 23 of 36 specimens (63.9%) derived from re-intervention, whereas type 2 (GCD2) and unspecified granular dystrophy demonstrated lower proportions with 6 of 18 specimens (33.3%) and 2 of 25 specimens (8.0%), respectively. For lattice corneal dystrophy (LCD), the re-intervention rate was 32.8% (21/64) per specimen and was even higher when assessed per patient, at 40.9% (18/44). Conversely, macular corneal dystrophy (MCD) and Epithelial Basement Membrane Dystrophy (EBMD) showed the lowest re-intervention rates. For MCD, this was 4.6% (3/65) per specimen and 6.1% (3/49) per patient. For EBMD, the proportion was 4.0% for both specimens (1/25) and patients (1/25).

In contrast, no specimens were associated with surgical re-intervention in cases of epithelial recurrent erosion dystrophy (ERED, *n* = 2 specimens in the initial cohort), Franceschetti corneal dystrophy (FRCD, *n* = 2), subepithelial mucinous corneal dystrophy (SMCD, *n* = 5), Reis-Bücklers corneal dystrophy (RBCD, *n* = 4), congenital stromal dystrophy (CSCD, *n* = 4), posterior amorphous corneal dystrophy (PACD, *n* = 1), Pre-Descemet dystrophy (PDCD, *n* = 1), posterior polymorphous corneal dystrophy (PPCD, *n* = 12), congenital hereditary endothelial dystrophy (CHED, *n* = 2). Likewise, no re-interventions were observed in unspecified epithelial (*n* = 7), stromal (*n* = 2), or endothelial (*n* = 16) corneal dystrophies, or in dystrophies with unspecified localisation (*n* = 2).

### Surgical procedures prior to re-intervention

The surgical procedures preceding re-intervention varied according to dystrophy type and anatomical localisation, as illustrated in Fig. 2. All EBMD, MECD and Lisch epithelial corneal dystrophy specimens obtained after re-intervention were linked to prior MSK or PTK. However, GDLD, also an epithelial and subepithelial dystrophy, demonstrated a broader surgical spectrum: among the 25 re-intervention specimens, 13 followed LimboKP (52.0%), one followed PKP (4.0%), 6 followed MSK (24.0%), and 5 followed PTK (20.0%).

Epithelial-stromal dystrophies, such as GCD, LCD and Thiel–Behnke corneal dystrophy, demonstrated also varying surgical approaches prior to re-intervention. In GCD1, 2 of 23 re-intervention specimens followed LimboKP (8.7%), 10 PKP (43.5%) and 11 PTK (47.8%). For GCD2, 4 of 6 followed PKP (66.7%) and 2 PTK (33.3%). Among GCD of unspecified type, 2 of 2 were associated with PKP (100.0%). In LCD, 2 specimens followed LimboKP (9.5%), 17 PKP (81.0%), and 2 PTK (9.5%). For Thiel–Behnke corneal dystrophy one specimen each followed LimboKP (50.0%) and PKP (50.0%). The single Gly623Asp-associated corneal dystrophy re-intervention specimen, referring to a *TGFBI*-related dystrophy caused by substitution of glycine by aspartic acid at amino acid position 623 [13], followed PTK (100.0%). Cases of unspecified epithelial-stromal dystrophies had one specimen linked to PKP (100.0%).

Stromal dystrophies exhibited distinctive patterns in relation to surgical re-intervention. In MCD, all 3 re-intervention specimens followed PTK (100.0%). Similarly, in Schnyder corneal dystrophy (SCD), both re-intervention specimens followed a minimally invasive technique, one specimen followed MSK (50.0%) and one PTK (50.0%).

Endothelial dystrophies, including posterior polymorphous corneal dystrophy, congenital hereditary endothelial dystrophy and unspecified endothelial corneal dystrophies, as well as other dystrophies mentioned in the section *Proportion of Surgical Re-Interventions* above, showed no specimens derived from surgical re-intervention.

## Discussion

This study highlights the variability in surgical re-intervention patterns across non-Fuchs corneal dystrophies in a large histopathology-based dataset spanning eight decades. The results are consistent with existing literature regarding recurrence and provide a novel insight into the performance of different surgical approaches.

The nearly balanced sex distribution in the present re-intervention series (48.3% female, 51.7% male) suggests no clear sex-related pattern. This is consistent with the predominantly autosomal inheritance of most corneal dystrophies included in this study, for which a strong sex predilection is generally not expected [4]. Nevertheless, given the small specimen and patient counts within individual dystrophy subgroups, no definitive conclusions can be drawn regarding sex-related differences within specific dystrophy types.

In our archive, the highest proportion of re-interventions were of epithelial or epithelial-stromal origin, consistent with previous reports that emphasise their tendency to recur following surgery [14, 15]. Among all, GDLD, which is associated with an abnormal increase in the permeability of the corneal epithelium [16], exhibited the highest proportion of re-intervention in this study, including multiple cases despite prior LimboKP. This finding highlights GDLD’s progressive and challenging clinical course, as previously described in the literature [17]. Stromal dystrophies, such as MCD (3/65 specimens, 3/49 patients) and SCD (2/11 specimens, 2/10 patients) exhibited low re-intervention rates in our dataset. Non-Fuchs endothelial dystrophies, such as posterior polymorphous corneal dystrophy, congenital hereditary endothelial dystrophy, and other non-specified endothelial corneal dystrophies showed no specimens derived from recurrence in our study, suggesting effective management with the initial interventions, including solely PKP and DMEK [11]. However, the absence of recurrence must be interpreted cautiously, given low case counts (30 specimens). While, to our knowledge, no cases of congenital hereditary endothelial dystrophy recurrence have been reported in the literature [18, 19], recurrence of posterior polymorphous corneal dystrophy has already been described due to proliferation and migration of pathological endothelium from the host periphery into the donor graft [8, 20].

Surgical re-interventions were observed in cases managed with minimally invasive approaches. For MSK, re-intervention was primarily associated with epithelial and subepithelial dystrophies like Lisch Epithelial Corneal Dystrophy and Meesmann Corneal Dystrophy (MECD), consistent with previous findings [21, 22]. PTK, while frequently employed for epithelial-stromal dystrophies with anterior opacities, exhibited notable re-intervention rate, particularly in granular dystrophies, where 13 of 31 re-intervention-associated specimens followed this technique, including 11 of 23 GCD1 specimens and 2 of 6 GCD2 specimens, aligning with reports highlighting elevated recurrence rates after PTK for these dystrophies [23]. As in EBMD, MECD and Lisch epithelial corneal dystrophy, re-intervention specimens from MCD and SCD were exclusively linked to superficial procedures (MSK and PTK).

This finding that re-interventions in true stromal dystrophies were only linked to superficial procedures supports earlier reports that full-thickness grafting is a highly durable treatment for these dystrophy types [14, 24]. While recurrence after PKP is rare, it cannot be entirely excluded, as some studies have reported recurrent cases in both MCD and Schnyder dystrophy even after PKP [7, 23, 25]. A possible mechanism involves migration of diseased host keratocytes or abnormal glycoproteins into the donor tissue, potentially contributing to recurrence [26, 27]. This rarity of recurrence is likely explained by the stromal aetiology, in which diseased keratocytes are effectively replaced by the donor cornea. More recent findings further implicate the corneal endothelium in MCD pathogenesis, as CHST6 (carbohydrate sulfotransferase 6), the causative gene for MCD, has been found to be active not only in stromal cells but also in the corneal endothelium [28]. Consequently, endothelium replacement during PKP may contribute significantly to preventing recurrence. This supports the preference for PKP over DALK in MCD [29], a conclusion also reinforced by Cheng et al., who found that PKP was associated with both a lower recurrence rate (17.5% vs. 42.9%) and delayed onset of recurrence compared with DALK [7]. Although MSK, PTK, and DALK offer advantages such as reduced invasiveness, their limited treatment depth predisposes to recurrence once deeper stromal or endothelial layers accumulate dystrophic deposits. This underscores the need for tailored postoperative strategies and closer follow-up.

The absence of DALK in our re-intervention series for other stromal dystrophies, such as GCD and LCD, also merits attention. While DALK is an accepted option for these *TGFBI*-associated dystrophies [30–32], this choice involves a critical trade-off regarding the risk of recurrence [23, 33, 34]. A study by Lewis et al. [35] on GCD clearly demonstrated that recurrence-free survival was significantly longer after PKP (median 13.7 years) compared to DALK (median 3.2 years). The scientific basis for this high recurrence is debated, with evidence supporting two potential mechanisms: a primary epithelial origin, where the graft-host interface acts as a scaffold for new deposits [36], and an independent stromal origin from residual defective host keratocytes retained in the posterior bed [34, 37]. The DALK procedure is anatomically vulnerable to both potential sources, as it retains the host posterior stroma and creates a graft-host interface susceptible to deposits from the repopulating host’s defective epithelium. This provides a strong rationale for our centre’s preference for full-thickness grafting, a decision aimed at maximising long-term, recurrence-free graft survival. The fact that many specimens in our eight-decade cohort predate the widespread adoption of DALK likely also explains its absence.

Nevertheless, PKP is not uniformly effective across all dystrophies. Despite the advantages of PKP, our findings indicate that recurrence remains a challenge in epithelial-stromal dystrophies. In GCD (all types), 16 of 31 specimens followed PKP (51.6%), including 10 of 23 specimens in GCD1 (43.5%), 4 of 6 in GCD2/Avellino (66.7%), and 2 of 2 (100.0%) in GCD of unspecified type. In LCD, PKP was the most frequent preceding procedure, accounting for 17 of 21 specimens. Despite PKP being widely used in these conditions, our data indicate that a considerable number of patients eventually require repeat surgery. This is exemplified in LCD, where the per-patient re-intervention rate (40.9%) was even higher than the per-specimen rate (32.8%), indicating that multiple repeat surgeries were often necessary in the same individuals. The findings indicate that recurrence after PKP is not uncommon in these dystrophy types and highlight the limited long-term efficacy of full-thickness grafting in epithelial-stromal dystrophy [38].

Recognising these limitations, Sundmacher, Spelsberg, and Reinhard at the Eye Hospital Düsseldorf implemented the LimboKP method in 1995 with the aim of minimising the recurrence of these dystrophies within grafts [38]. Since 2003, LimboKP has become a frequently utilised method in Freiburg as well [11]. Spelsberg et al. demonstrated that LimboKP was associated with reduced recurrence rates in GCD and LCD [15]. These findings are consistent with our results, as only two specimens each were linked to recurrences in GCD (out of 31 specimens) and in LCD (out of 21 specimens) following LimboKP. Notably, however, 13 of the GDLD re-intervention specimens in our study followed LimboKP, indicating that even this targeted approach may not consistently prevent recurrence in this dystrophy type. This underscores the high clinical burden of GDLD, where the need for re-intervention remains frequent at the patient level, with 25 re-intervention specimens originating from just 5 patients and 80.0% (4 out of 5 patients) requiring bilateral repeat surgeries over time. Furthermore, patients with GDLD were younger at the first surgical re-intervention (mean: 32.2 ± 10.3 years; range: 19 to 45 years) than those in the overall re-intervention series (mean: 54.6 ± 16.1 years) and patients with other frequently represented dystrophies, such as GCD across all subtypes (mean: 54.8 ± 17.2 years) and LCD (mean: 61.0 ± 10.2 years). This highlights the early-manifestation, progressive course, and highly relapsing nature of this disease. Definitive conclusions regarding the efficacy of LimboKP remain limited due to the retrospective design of this study, which only determined whether a specimen was associated with surgical re-intervention and which surgical technique had been performed prior to it. Importantly, our findings indicate that re-interventions remain necessary in some cases even after LimboKP for GDLD, Thiel–Behnke corneal dystrophy, LCD, and GCD, underscoring the importance of rigorous postoperative monitoring and tailored follow-up strategies for managing these epithelial-origin dystrophies.

This study has limitations, the most important of which is that this is a single‑centre retrospective study. The eight-decade timeline adds variability due to evolving diagnostic criteria, surgical techniques, and nomenclature, potentially affecting data consistency. Despite reclassification efforts, diagnostic drift cannot be fully excluded. In addition, although all included re-intervention specimens showed histopathological changes compatible with the underlying corneal dystrophy, consistent with recurrence, the exact clinical indication for repeat surgery could not always be reconstructed with certainty and may have been multifactorial. Given the retrospective nature of the study, some cases of surgical re-intervention may have remained undetected due to incomplete documentation of earlier recurrences or limited follow-up data. At our centre, corneal surgical tissue is routinely submitted for histopathological examination, making the likelihood of missed internal re-intervention specimens low. However, this cannot be assumed for externally referred cases, for which longitudinal records and information on procedures performed elsewhere were not consistently available. Therefore, we could not determine the number of re-interventions without specimen submission, nor can we fully exclude additional interventions for which no tissue was submitted to our archive. This may have led to an underestimation of the clinical re-intervention rate. Additionally, our statistical analysis is descriptive, which is further constrained by the low overall case numbers for several extremely rare non-Fuchs corneal dystrophy types. Although temporal spacing between archived same-eye re-intervention specimens could be described for eyes with multiple archived specimens, the retrospective, specimen-based design precluded formal survival analysis. Such analysis would have required complete longitudinal follow-up of all eyes, including those without further archived re-intervention specimens, and would have provided more comprehensive insights into recurrence timing and long-term surgical durability. This limitation highlights the need for prospective studies with standardised follow-up.

This study also has several notable strengths. Its foundation is a comprehensive, eight-decade histopathological archive, which affords extensive longitudinal data across nearly the entire spectrum of non-Fuchs corneal dystrophies and ensures diagnostic certainty through histopathology for each case. A key methodological strength is the dual analysis on both a per-specimen and per-patient basis. This approach, built on reporting the underlying counts for both specimens and unique patients, enables a far more detailed interpretation than a single percentage could offer. It reveals that the high specimen-to-patient ratio in certain dystrophies, such as GDLD, is itself a finding, that directly reflects the highly relapsing nature of the disease and quantifies the substantial treatment burden imposed on affected individuals.

In conclusion, the findings indicate that surgical re-intervention remains a clinically relevant concern in non-Fuchs corneal dystrophies. Importantly, the need for re-intervention is not uniform but rather intrinsically linked to the dystrophy type and the surgical approach. Epithelial-origin dystrophies, particularly gelatinous drop-like dystrophy, exhibited the highest re-intervention rates, even after targeted procedures such as LimboKP. Furthermore, this study provides a nuanced perspective on penetrating keratoplasty, which, while a durable treatment for purely stromal conditions like macular dystrophy, is frequently followed by recurrence in *TGFBI*-associated dystrophies. These results highlight the limitations of current surgical approaches for recurrence-prone dystrophies and emphasise the necessity for type-specific surgical strategies, the development of more effective techniques to prevent recurrence, and structured long-term follow-up protocols adapted to individual risk.

## Summary

### What was known before

- Recurrence following surgery for corneal dystrophies is an established clinical challenge, with the risk known to vary by dystrophy type.
- Prior studies have often focused on recurrence in single dystrophy types, but large-scale, real-world data quantifying the actual need for surgical re-intervention across a broad range of dystrophies were lacking

### What this study adds

- This is the largest and longest-term study to date to quantify the surgical re-intervention burden across a wide spectrum of non-Fuchs dystrophies, based on an eight-decade histopathologically confirmed cohort covering nearly all IC3D-classified entities.
- The study demonstrates a stark contrast in long-term surgical outcomes: while no re-interventions were observed following penetrating keratoplasty for true stromal dystrophies, a high clinical burden of repeat surgery was identified in TGFBI-associated and gelatinous dystrophies, often involving multiple procedures in the same patients, and for the latter, even after targeted procedures like limbo-keratoplasty.

## Data Availability

The datasets analysed during the current study are not publicly available due to patient confidentiality and data protection requirements, but are available from the corresponding author on reasonable request, subject to institutional and ethical approval.
